# DNA Demethylation Switches Oncogenic ΔNp63 to Tumor Suppressive TAp63 in Squamous Cell Carcinoma

**DOI:** 10.3389/fonc.2022.924354

**Published:** 2022-07-14

**Authors:** Zuzana Pokorna, Vaclav Hrabal, Vlastimil Tichy, Borivoj Vojtesek, Philip J. Coates

**Affiliations:** ^1^ Research Center of Applied Molecular Oncology (RECAMO), Masaryk Memorial Cancer Institute, Brno, Czechia; ^2^ Department of Experimental Biology, Faculty of Science, Masaryk University, Brno, Czechia

**Keywords:** ΔNp63, TAp63, alternative promoter usage, squamous cell carcinoma, keratinocytes, DNA methylation, decitabine

## Abstract

The *TP63* gene encodes two major protein variants; TAp63 contains a p53-like transcription domain and consequently has tumor suppressor activities whereas ΔNp63 lacks this domain and acts as an oncogene. The two variants show distinct expression patterns in normal tissues and tumors, with lymphocytes and lymphomas/leukemias expressing TAp63, and basal epithelial cells and some carcinomas expressing high levels of ΔNp63, most notably squamous cell carcinomas (SCC). Whilst the transcriptional functions of TAp63 and ΔNp63 isoforms are known, the mechanisms involved in their regulation are poorly understood. Using squamous epithelial cells that contain high levels of ΔNp63 and low/undetectable TAp63, the DNA demethylating agent decitabine (5-aza-2’-deoxycytidine, 5-dAza) caused a dose-dependent increase in TAp63, with a simultaneous reduction in ΔNp63, indicating DNA methylation-dependent regulation at the isoform-specific promoters. The basal cytokeratin *KRT5*, a direct ΔNp63 transcriptional target, was also reduced, confirming functional alteration of p63 activity after DNA demethylation. We also showed high level methylation of three CpG sites in the *TAP63* promoter in these cells, which was reduced by decitabine. DNMT1 depletion using inducible shRNAs partially replicated these effects, including an increase in the ratio of *TAP63*:*ΔNP63* mRNAs, a reduction in ΔNp63 protein and reduced *KRT5* mRNA levels. Finally, high DNA methylation levels were found at the *TAP63* promoter in clinical SCC samples and matched normal tissues. We conclude that DNA methylation at the *TAP63* promoter normally silences transcription in squamous epithelial cells, indicating DNA methylation as a therapeutic approach to induce this tumor suppressor in cancer. That decitabine simultaneously reduced the oncogenic activity of ΔNp63 provides a “double whammy” for SCC and other p63-positive carcinomas. Whilst a variety of mechanisms may be involved in producing the opposite effects of DNA demethylation on TAp63 and ΔNp63, we propose an “either or” mechanism in which *TAP63* transcription physically interferes with the ability to initiate transcription from the downstream *ΔNP63* promoter on the same DNA strand. This mechanism can explain the observed inverse expression of p63 isoforms in normal cells and cancer.

## Introduction

The *TP63* gene codes for two major protein variants using transcripts produced from two separate promoters. Unlike TAp63, which is transcribed from the upstream promoter (P1) and contains an N-terminal p53-like transactivation sequence, ΔNp63 is initiated at a downstream promoter (P2) and the protein lacks the N-terminal transactivation domain. Thus, ΔNp63 was originally thought to act as a transcriptional repressor, but was subsequently shown to contain an alternative transactivation domain, inducing expression of target genes involved in proliferation, survival, adhesion and differentiation of stratified epithelial cells and tissues such as breast, prostate and urothelium (1, 2).

TAp63 has tumor suppressor roles that reflect its p53-like activities of inducing apoptosis (3) or senescence (4), and inhibiting metastasis (5, 6). In contrast, ΔNp63 promotes tumorigenesis (7, 8) and resistance to cytotoxic therapies (9). The tumor suppressor properties of TAp63 stimulated attempts to induce TAp63 as a therapeutic strategy, producing reduced cell viability and enhanced response to therapy (10). In particular, because TAp63 has p53-like properties, this approach is a viable option for replacing p53 tumor-suppressive activities in tumors with p53 mutation (10, 11). Similarly, reducing the oncogenic activity of ΔNp63 causes tumor-suppressive activities for tumors that overexpress this protein (12, 13).

In human cancers, ΔNp63 is commonly overexpressed in squamous cell carcinoma (SCC), whereas some B-cell lymphomas/leukemias express TAp63 (14, 15). These patterns of p63 isoforms in malignancy reflect their normal tissue expression patterns - TAp63 but not ΔNp63 is present in oocytes and lymphocytes, whereas ΔNp63 is the only form present in most adult squamous epithelia (1, 2, 16). In tumors that overexpress ΔNp63, the level of TAp63 associates with improved patient survival in SCC of the cervix and triple-negative breast cancers, in keeping with a tumor suppressor role of TAp63 in these and other cancers (14, 17–20). However, how the ΔNp63 and TAp63 isoforms are regulated to produce their tissue-specific expression patterns and their dysregulation in cancer is not known. Understanding the pathways involved in TAp63 and ΔNp63 regulation is therefore important to enable manipulation of their levels for cancer treatment. The aim of a p63-based therapeutic approach for tumors with high levels of ΔNp63 is to either decrease ΔNp63 or to increase TAp63, either of which can reduce tumor cell growth on their own (10–13). Here, we investigated DNA methylation at the *TP63* gene locus as a potential regulator of p63 isoform transcription. This notion was based in part on evidence from studies of leukemic cells (TAp63-positive, ΔNp63-negative), where an inverse correlation exists between *TP63* mRNA levels and methylation at the P1 *TAP63* promoter (21, 22). In addition, hypomethylation at the P2 *ΔNP63* promoter in SCC (23–25), provides evidence for P2 promoter methylation as a repressive mechanism for ΔNp63. Thus, we investigated whether the DNA methyltransferase inhibitor (DNMTi) decitabine (5-Aza-2’-deoxycytidine; 5dAza), can activate TAp63 in SCC by reducing P1 methylation without influencing ΔNp63 levels from the already demethylated P2 promoter. Unexpectedly, we found that decitabine not only increased TAp63, but also caused a concomitant reduction in ΔNp63 protein and mRNA. Genetic depletion of DNMT1 partially recapitulated these results. The data show for the first time that *TP63* transcription can be switched from oncogenic ΔNp63 to tumor suppressor TAp63 and that this can be achieved using a clinically approved DNA demethylating agent.

## Material and Methods

All reagents and chemicals were obtained from Sigma-Aldrich (St. Louis, MO, USA) unless stated otherwise. Control treatments were performed using the same volume of the corresponding solute (DMSO or water for decitabine or doxycycline, respectively).

### Cell Culture and Treatments

FaDu (human pharynx squamous cell carcinoma) and SCC-25 (human squamous cell carcinoma of the tongue) were obtained from the American Type Culture Collection (ATCC, Manassas, VA, USA). Non-transformed squamous HaCaT cells (spontaneously immortalized human keratinocytes) were obtained from DKFZ (Heidelberg, Germany). FaDu and HaCaT cells were cultured in high glucose Dulbecco’s modified Eagle’s medium (DMEM) with 10% fetal bovine serum (FBS), 1% sodium pyruvate, and penicillin/streptomycin (all from Gibco, Thermo Fisher Scientific, MA, USA) at 37°C in 5% CO_2_. SCC-25 cells were cultured in DMEM/Nutrient Mixture F-12 (DMEM/F-12, Gibco) with 10% FBS, 0.4 μg/ml hydrocortisone (Lonza, Basel, Switzerland), 1% sodium pyruvate, and penicillin/streptomycin. Cells were grown to 40-70% confluency before treatment according to the type and length of the experiment. Cell viability was determined using Resazurin (see the supplementary material for details). Samples used for Western blotting and RT-qPCR were analyzed in at least three biological triplicates.

### Inducible DNMT1 Knockdown Cell Lines

Five individual TET-inducible TRIPZ plasmids containing shRNAs targeting *DNMT1* were obtained from Horizon Discovery (RHS4740-EG1786, Cambridge, UK). Plasmid DNAs were isolated using a Plasmid Maxi Kit (12162, Qiagen, Hilden, Germany) and used to produce viral particles in HEK293FT cells (ATCC). Lentiviruses were collected 48 and 96 h after transfection and transduced into HaCaT, FaDu and SCC-25 cells as described previously (12). Medium was replaced after 24 h, and selection in puromycin (1 µg/ml for HaCaT and FaDu and 0.05 µg/ml for SCC-25) started after a further 24 h. Medium containing puromycin was replaced every three days. Puromycin-resistant cells were expanded, shRNAs were induced with 2 µg/ml doxycycline and DNMT1 was assessed by Western blotting. Cell populations showing DNMT1 downregulation were single-cell sorted (BD FACS Aria III, Wokingham, Berks., UK) into 96-well plates and two individual clones were prepared for each cell line after further puromycin selection. Individual clones were re-tested for doxycycline-inducible DNMT1 knockdown by Western blotting. SCC-25 cell clones died during puromycin selection and no stable clones were obtained. Stable cell lines were obtained for HaCaT and FaDu and were routinely cultured in DMEM with 10% FBS containing 1 µg/ml puromycin. Doxycycline was added at 2 µg/ml final concentration to induce shRNA-mediated depletion of DNMT1 and the medium was replaced with freshly prepared medium every 24 h. Cells were analyzed after four or six days of continuous doxycycline and compared to the same cells grown in the absence of doxycycline.

### DNA Methylation at the TAP63 Promoter

To investigate whether methylation at the P1 promoter could be a mechanism for regulating *TAP63* transcription, we searched CpG methylation profiles in the ENCODE project (https://www.encodeproject.org/) (26) in lymphocyte/leukemia cell lines that may express TAp63 and in epithelial cells that may express ΔNp63. To measure CpG methylation at the identified sites, cells were harvested with trypsin and genomic DNA isolated (QIAamp, Qiagen, Manchester, UK). Bisulfite conversion of 500 ng DNA was performed using EZ DNA methylation (Zymo, Irvine, CA, USA) according to the manufacturer’s instructions. Bisulfite converted PCR primers (Generi Biotech, Hradec Kralove, Czech Republic) were designed according to MethPrimer 2.0 (urogene.org/cgi-bin/methprimer2/MethPrimer.cgi) (27) (**Supplementary Table S1**) to amplify a 134 bp region beginning 111 bp upstream of the *TAP63* transcription start site and containing three CpG sites. PCR was performed using Taq polymerase (Invitrogen, ThermoFisher Scientific, Waltham MA, USA): 95°C for 30 s and 40 cycles of 95°C for 15 s, 50°C for 20 s and 68°C for 90 s, followed by a final extension at 72°C for 3 min. PCR products were analyzed on 1.5% agarose gels and purified (QIAquick gel extraction kit, Qiagen) for Sanger sequencing (Eurofins Genomics, Benesov, Czech Republic) using the forward or reverse PCR primer. CpG methylation was calculated as described (28, 29) using the peak heights of the bisulfite modified nucleotide at the CpG sites and measuring the average peak heights on either side.

To investigate methylation levels in clinical SCC samples, we retrieved publicly available data from the Gene Expression Omnibus repository (https://www.ncbi.nih.gov/geo/) for three independent patient cohorts; TCGA analysis of DNA methylation for lung SCC (GSE 68825, updated 2019); a separate cohort of lung SCC samples [GSE66045 (30)] and patients with oropharyngeal SCC [GSE124464 (31)]. All cohorts included matched normal tissue samples. Further details are provided in the supplementary materials.

### Western Blotting

See the supplementary material for details. Total proteins were separated using SDS-PAGE, blotted onto nitrocellulose and the membranes cut into horizontal strips using the molecular weight markers. Individual strips of the same blot were used to detect different proteins. Target proteins were identified using the mouse monoclonal antibodies ΔNp63 1.1 and TAp63 4.1 for p63 isoforms, as described previously (17, 32) and rabbit antibodies to DNMT1 (clone D63A #5032) and γ-H2AX (#9718, both from Cell Signaling Technology, Danvers, MA, USA). β-actin (clone C4, sc-47778, Santa Cruz Biotechnology, Dallas, TX, USA) was used as loading control. Proteins were detected with peroxidase-conjugated goat anti-mouse or anti-rabbit IgG (Jackson Immunoresearch, West Grove, PA, USA) and enhanced chemiluminescence (ECL, Amersham Pharmacia Biotech, Bucks, UK). Blots were quantified by densitometry using ImageJ (https://imagej.nih.gov/ij/) and normalized to β-actin.

### RNA Isolation and Reverse Transcription-Quantitative PCR

Total RNA was isolated using TRIzol and 500 ng were reverse transcribed using High Capacity cDNA Reverse Transcription (Applied Biosystems, ThermoFisher Scientific). PCR primers were obtained from Generi Biotech (**Supplementary Table S1**) and PCR was performed on a Fast Real-Time PCR System with Sybrgreen (Applied Biosystems): 95°C for 3 min, 50 cycles of 95°C for 5 s and 60°C for 25 s. At least three biological replicates were performed, and each cDNA sample was analyzed in technical triplicates. Mean cycle threshold (Ct) values were normalized to β-actin (*ACTB*) and transformed into relative mRNA levels (33).

### Statistical Analysis

Data were tested for normal distribution using Shapiro-Wilk tests with significance level (α) = 0.05 and examining skewness and excess kurtosis (https://www.statskingdom.com/shapiro-wilk-test-calculator.html). No experimental dataset showed a significant departure from normality. Therefore, these data are presented as mean ± SEM and statistical significance was determined using unpaired 2-tailed t-tests against control values. In **Figure 9**, where data showed a significant deviation from normality, Mann-Whitney test was used. p < 0.05 was considered significant.

## Results

### Decitabine Increased TAp63 mRNA and Protein Levels in SCC

**Figure 1:**
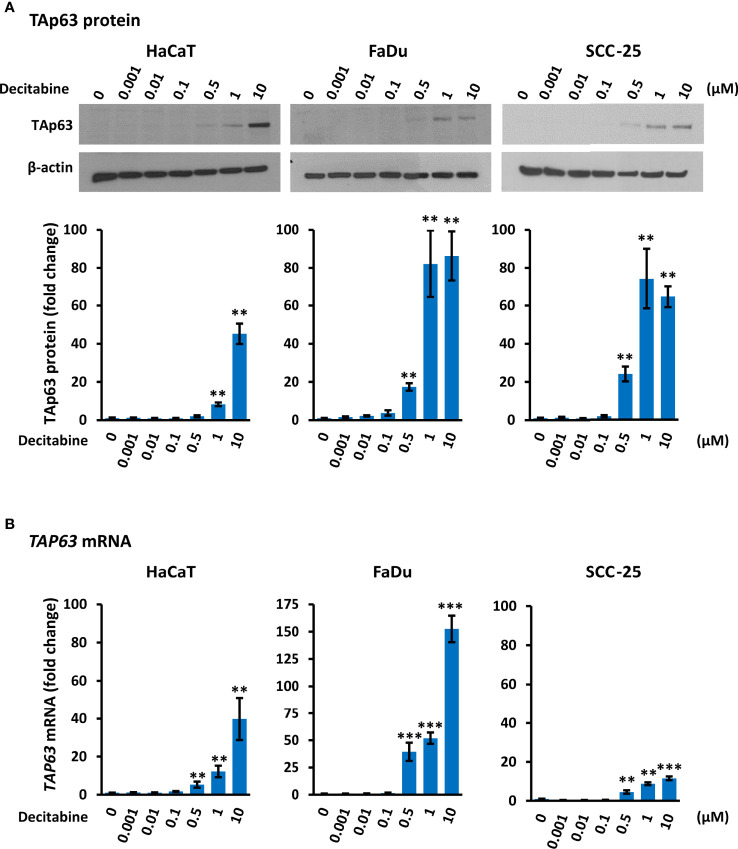
*Decitabine increases TAp63 protein and mRNA levels in squamous cells*. **(A)** Representative Western blots of TAp63 in HaCaT, FaDu and SCC-25 cells cultured in the presence of the indicated concentration of decitabine for four days. β-actin is shown as loading control. Densitometry data normalized to β-actin are shown below, indicating fold change ± SEM compared to control cells (0, DMSO only; n = 3 for each dose in each cell line). **(B)** *TAP63* mRNA levels normalized to *ACTB* mRNA (n = 3 to 5 biological replicates, each with technical triplicates). Data are shown as fold change ± SEM compared to control cells (0, DMSO only). Note the different *y* axis range for FaDu cells. **p < 0.01; ***p < 0.001 compared to control.

Decitabine acts to prevent re-methylation of newly synthesized DNA and therefore progressively reduces DNA methylation over increased numbers of replicative cycles. By Western blotting, TAp63 was present at low or undetectable levels in untreated HaCaT, FaDu and SCC-25 cells, in keeping with previous observations in transformed and non-transformed squamous cells (16, 34). Treatment with decitabine at concentrations from 0.001 µM to 10 µM for four days increased TAp63 protein in all three cell lines in a dose-dependent manner at concentrations of 0.5 µM and above. Accurate quantitation of TAp63 in Western blots is difficult due to the very low signals in untreated cells but was calculated as representing 45-fold to 86-fold induction at 10 µM decitabine (p < 0.01 for each cell line) (**Figure 1A**). These protein data correspond to the low levels of *TAP63* mRNA under normal growth conditions and the dose-dependent increases after treatment with decitabine at 0.5 µM and higher concentrations in all three cell lines, rising to between 10-fold and more than 100-fold higher levels in the different cell lines after 10 µM decitabine (p < 0.01 for HaCaT and p < 0.001 for FaDu and SCC-25) (**Figure 1B**).

### Decitabine Decreased ΔNp63 mRNA and Protein Levels in SCC

**Figure 2:**
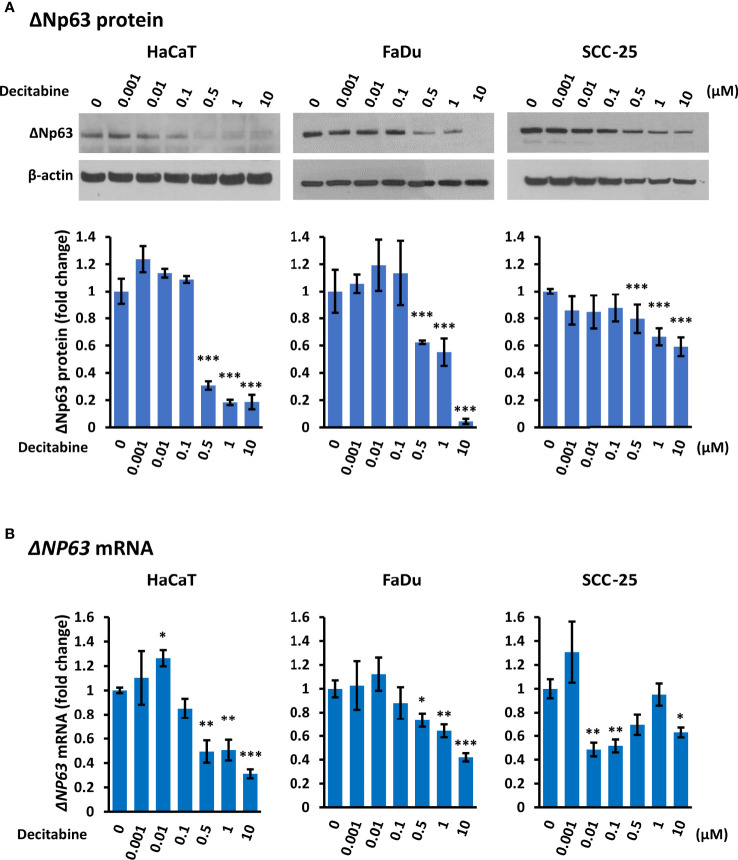
*Decitabine reduces ΔNp63 and mRNA levels in squamous cells.* **(A)** Representative Western blots and densitometric quantitation of ΔNp63 in HaCaT, FaDu and SCC-25 cells treated with the indicated concentrations of decitabine for four days. β-actin is shown as loading control. Densitometry data represent fold change ± SEM compared to untreated cells (0, DMSO only; n = 3 for each dose in each cell line) and normalized to β-actin. **(B)** *ΔNP63* mRNA levels normalized to *ACTB* mRNA (n = 3 to 5 biological replicates, each with technical triplicates). Data are shown as fold change ± SEM compared to untreated cells (0, DMSO only). *p < 0.05; **p < 0.01; ***p < 0.001 compared to control.

Conversely to TAp63, Western blotting showed that decitabine caused a dose-dependent reduction of ΔNp63 protein, with a 2-fold to 20-fold reduction after 10 µM decitabine for four days (**Figure 2A**). RT-qPCR also showed a 2-fold to 3-fold decrease in *ΔNP63* mRNA levels at higher decitabine concentrations, although low concentrations showed a slight increase in all cell lines, which was significant in HaCaT cells (p = 0.045 at 10 nM decitabine) (**Figure 2B**).

### Decitabine Decreased KRT5 mRNA Levels

**Figure 3:**
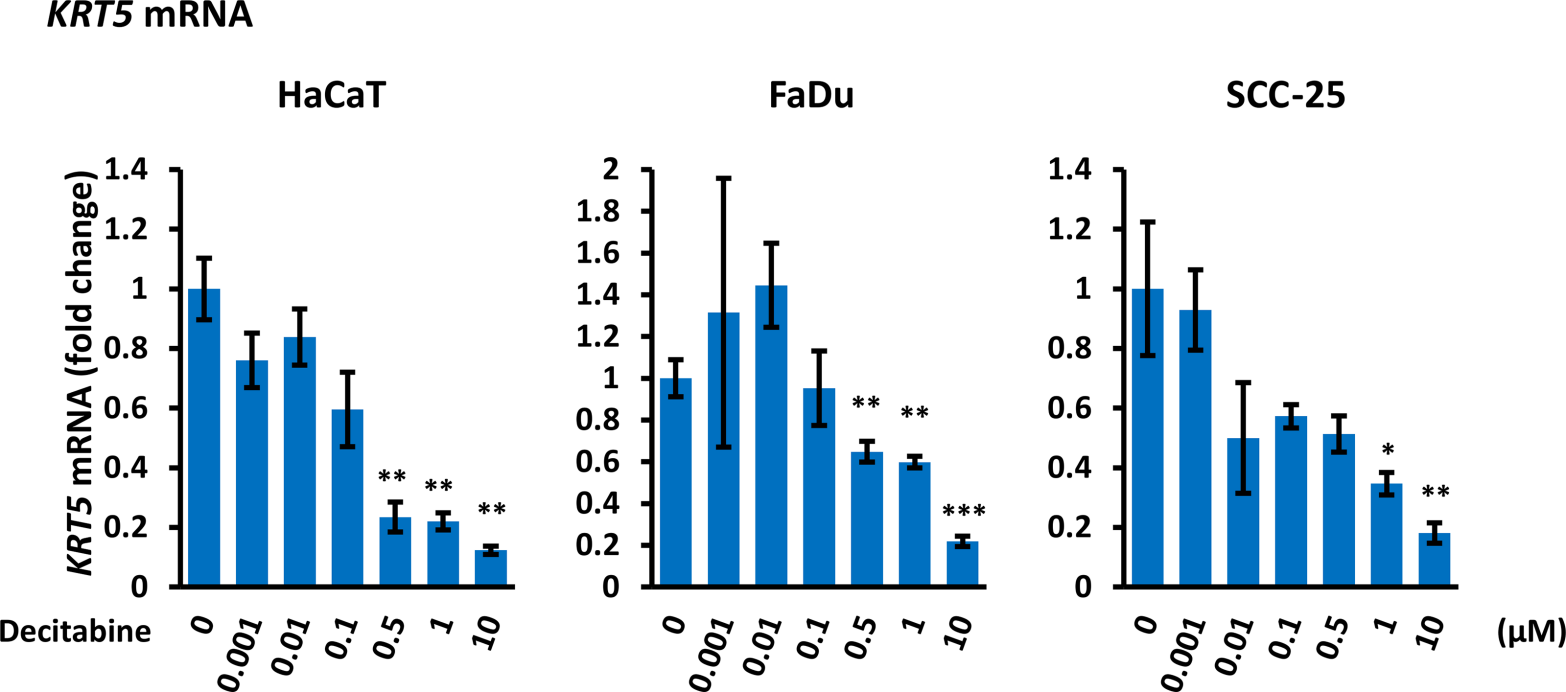
*Decitabine decreases KRT5 mRNA.* Cells were treated with the indicated concentrations of decitabine for four days and analyzed for *KRT5* mRNA by RT-qPCR. Data are shown as fold change ± SEM compared to control cells (0; DMSO only) and are normalized to *ACTB* mRNA (n = 3 biological replicates with technical triplicates for each concentration in each cell line). *p < 0.05; **p < 0.01; ***p < 0.001 compared to untreated cells.

To evaluate whether decitabine repression of ΔNp63 and induction of TAp63 influenced p63 transcriptional activity, we monitored the basal cytokeratins *KRT5* and *KRT14*, which are markers of undifferentiated basal squamous cells and are direct ΔNp63 target genes (35), and of *KRT1* and *KRT10* that are markers of squamous cell differentiation. Decitabine caused a reproducible and dose-dependent decrease in *KRT5* mRNA levels (5-fold to 10-fold reduction at the highest concentration of decitabine, p < 0.05 for each cell line) (**Figure 3**). Changes in other cytokeratins were inconsistent, with FaDu cells showing induction of *KRT1* and *KRT10* but no change in *KRT14* mRNA, whilst *KRT14* was decreased in SCC-25 without induction of *KRT1* or *KRT10* mRNAs (**Supplementary Figure S1**).

### Decitabine Reduced DNMT1 Levels and Caused Variable Levels of DNA Damage

**Figure 4:**
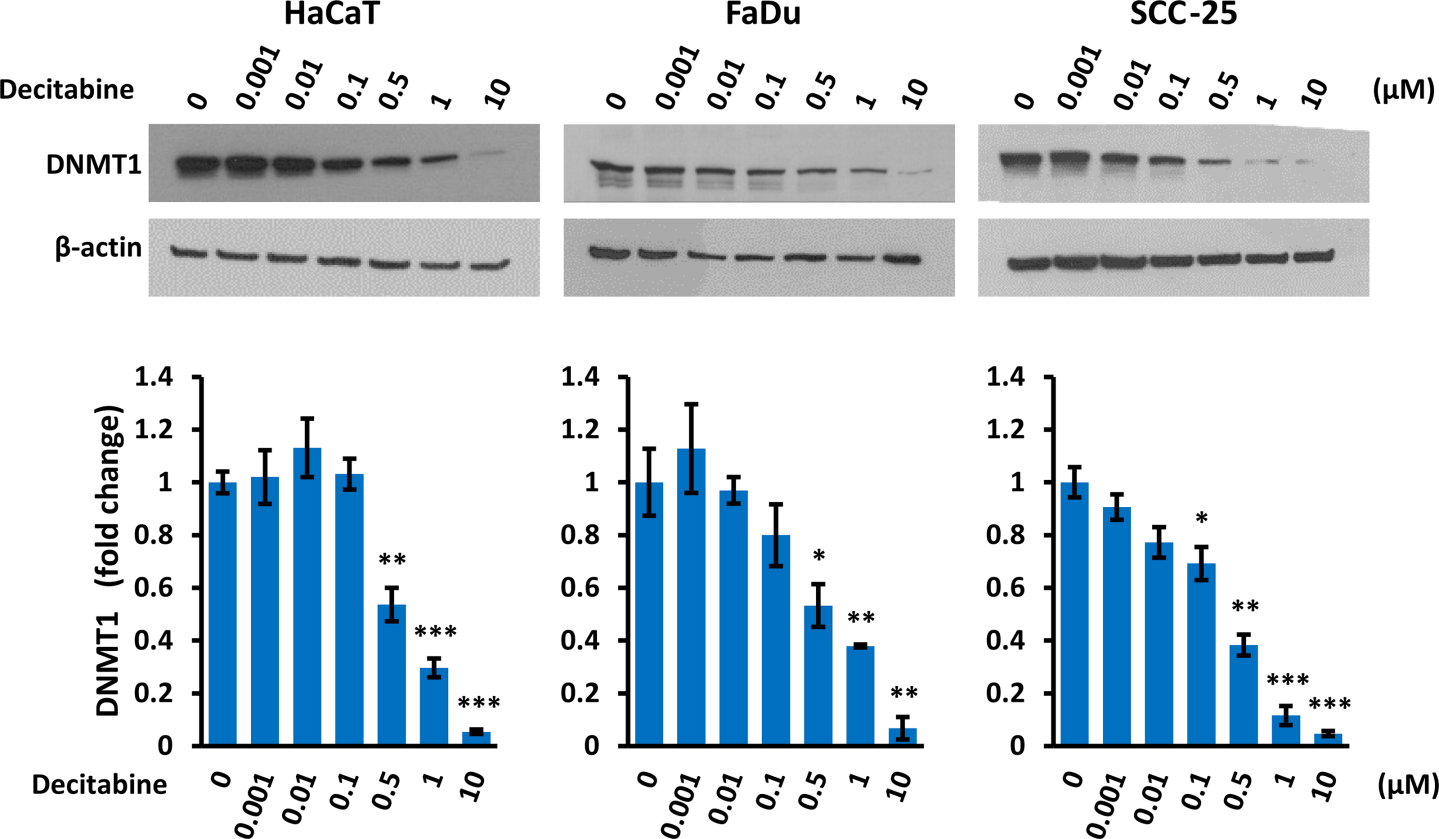
*Decitabine decreases DNMT1.* Representative Western blots and densitometry of DNMT1 in cells treated with the indicated concentration of decitabine for four days. Data are shown as fold change ± SEM compared to untreated cells (0; DMSO only) and are normalized to β-actin (n = 3 biological replicates for each concentration in each cell line). *p < 0.05; **p < 0.01; ***p < 0.001 compared to control cells.

Decitabine acts by covalent trapping of DNMTs on methylated DNA, leading to DNMT degradation and potentially causing DNA damage (36, 37). We confirmed that decitabine caused a dose-dependent reduction of soluble DNMT1 (**Figure 4**). In addition, high concentrations of decitabine increased the level of γ-H2AX as a marker of DNA double strand breaks (38) in FaDu cells which contain high levels of γ-H2AX under normal conditions, whereas HaCaT showed minimal γ-H2AX induction after decitabine and there was no apparent increase in SCC-25 cells (**Supplementary Figure S2**).

### DNMT1 Depletion Influences TAp63 and ΔNp63

**Figure 5:**
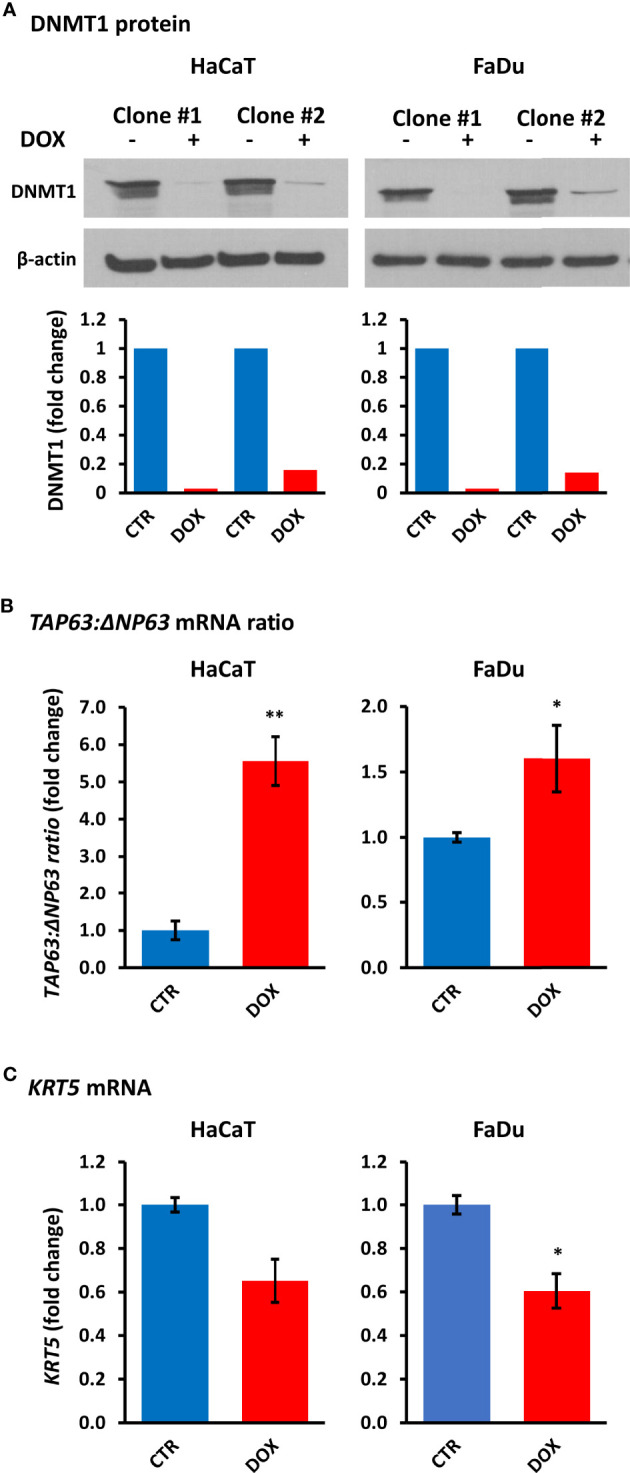
*DNMT1 depletion alters TP63 isoform mRNA levels and decreases KRT5 mRNA levels.* **(A)** Representative Western blots of DNMT1 in two independent clones of HaCaT and FaDu *DNMT1*-shRNA cells cultured for four days in the absence (–) or presence (+) of 2 µg/ml doxycycline (DOX). β-actin is shown as loading control. Densitometric quantitation of DNMT1 normalized to β-actin is shown below for each clone. **(B)** Relative changes in the ratio of *TAP63* to *ΔNP63* mRNA levels without induction (CTR) or after six days of 2 µg/ml doxycycline (DOX). Data were normalized to *ACTB* mRNA for each clone in each cell line and are shown as the average change for the two independent clones for each cell line. **(C)** *KRT5* mRNA levels in response to *DNMT1*-shRNA induction. Plots show fold change ± SEM compared to non-induced cells. n = 3 to 5 biological replicates; *p < 0.05; **p < 0.01.

To determine whether DNMT1 levels directly regulate the balance of TAp63 and ΔNp63 isoforms, we created cell lines with inducible *DNMT1*-shRNAs. Of five vectors tested, two (201896523 and 201895794) showed DNMT1 depletion after shRNA induction by doxycycline for four days (**Figure 5A**). Stable clones of HaCaT and FaDu cells were subsequently prepared (SCC-25 cells died during subsequent puromycin selection/maintenance and stable clones could not be produced). HaCaT and FaDu *DNMT1*-shRNA cells were induced with 2 µg/ml doxycycline for six days and analyzed for *TP63* isoform mRNAs, compared with the corresponding cells not exposed to doxycycline. These data showed an increase in *TAP63* and a decrease in *ΔNP63* mRNAs, depicted as the change in their ratio after DNMT1 depletion (**Figure 5B**) albeit with reduced effects compared to decitabine (compare with **Figure 1** and **Figure 2**). We also found a reduction in ΔNp63 levels by Western blotting, most noticeable in HaCaT cells but without a discernable increase in TAp63 protein, which remained undetectable by Western blotting (**Supplementary Figure S3**). However, *KRT5* mRNA was also reduced by *DNMT1*-shRNA, compatible with reduced *ΔNP63* activity (**Figure 5C**). The lack of measurable increase in TAp63 protein presumably reflects the relatively small effect of shRNA, such that TAp63 levels are not sufficiently increased to overcome the Western blot sensitivity threshold, whereas a decrease in ΔNp63 is visible due to its high endogenous levels.

### Altered TAp63 Levels Correlate With CpG Methylation at the TAp63 Promoter

**Figure 6:**
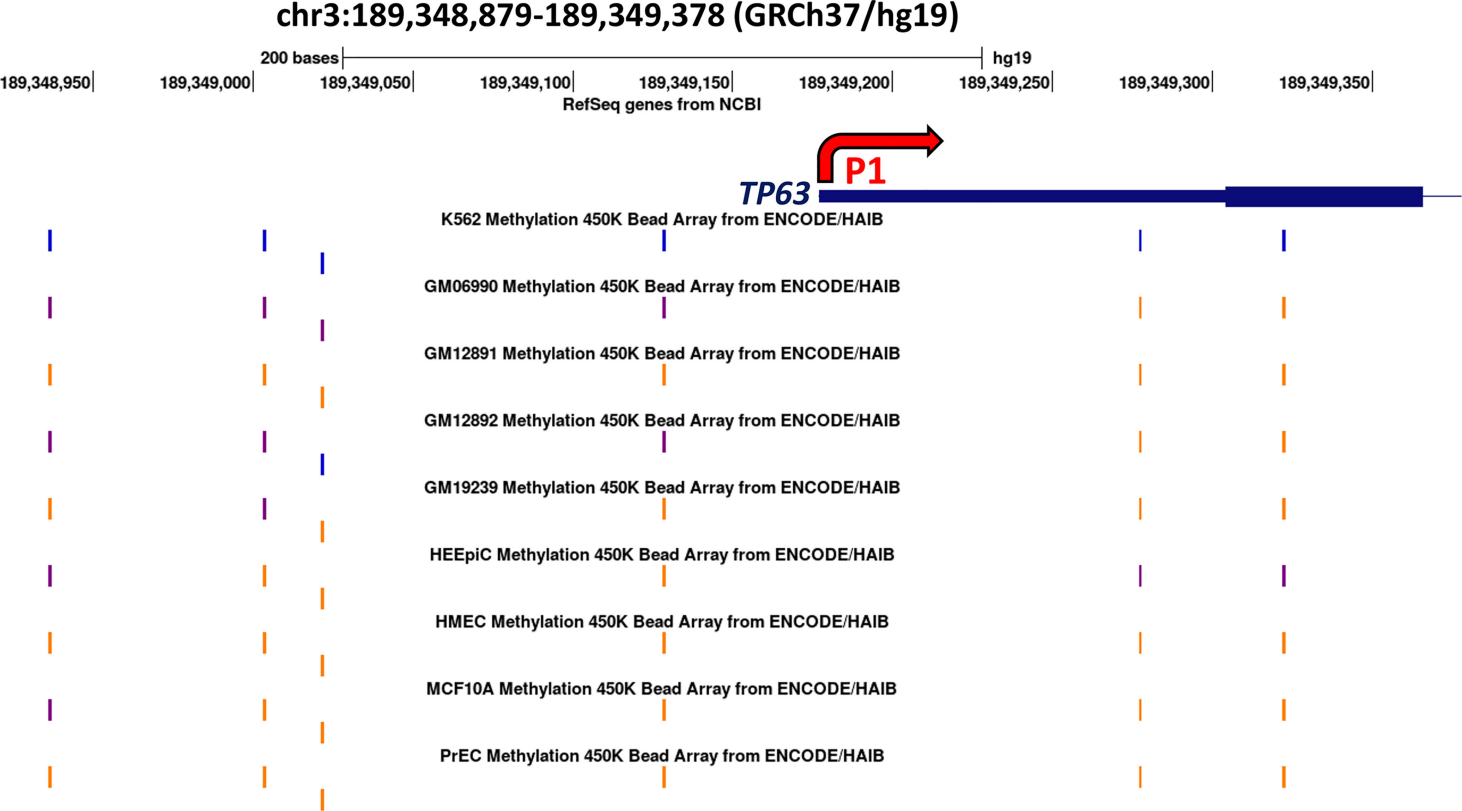
*Methylation array data surrounding the P1 promoter of the human TP63 gene for nine cell lines*. The blue line indicates the transcription start site of *TAp63* mRNA and the thicker blue line indicates the coding sequence of *TAP63* exon 1. The thin line indicates intron 1. Methylation levels of the six CpG sites present on the 450K bead array are shown; orange indicates high methylation (methylation score >= 600) blue indicates low methylation (<=200) and purple indicates intermediate methylation at each site on the array. Data are derived from UCSC (http://genome-euro.ucsc.edu/cgi-bin/hgGateway?redirect=manual) using DNA methylation data from ENCODE (26) overlaid onto the hg19 genome sequence. (K562, CML blast crisis; GM06990, GM12891, GM12892, and GM19239 are EBV-transformed B-lymphoblasts; HEEpiC, esophageal epithelial cells; HMEC, mammary epithelial cells; MCF10A, mammary epithelial cells (containing ER-Src); PrEC, prostate epithelial cells.

The above data suggest that TAp63 and ΔNp63 are regulated in squamous cells by DNA methylation. In particular, that DNMT inhibition increases TAp63 protein and mRNA implies that the P1 promoter is silenced by hypermethylation under normal conditions. To test this notion, we searched for evidence of differentially methylated CpG sites between cells that express TAp63 but not ΔNp63 and those that express ΔNp63 but not TAp63. We identified a series of CpG sites immediately upstream of P1 that show differential methylation profiles (**Figure 6**). These data show low/intermediate methylation of CpGs upstream of P1 in lymphocyte-derived cells that sometimes express TAp63 but not ΔNp63 (K562 lymphoblasts and EBV-transformed GM series lymphocytes), whilst epithelial cells that may express ΔNp63 but not TAp63 (HEEpiC, HMEC, MCF10A and PrEC) all show high methylation of the same CpGs. This association is not seen in CpGs downstream of the transcription start site (**Figure 6**), suggesting that CpG methylation immediately upstream of P1 may be involved in TAp63 regulation.

**Figure 7:**
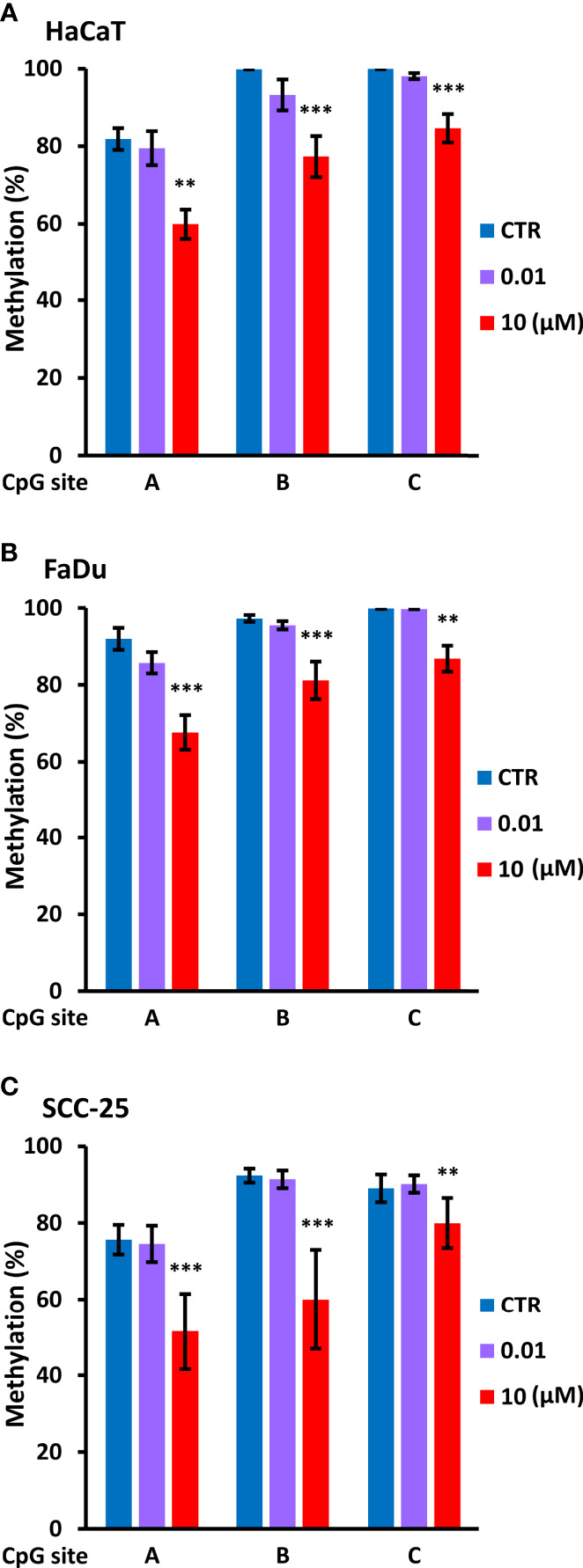
*Decitabine reduces DNA methylation at the TAp63 promoter.* The indicated cell lines were untreated (CTR, DMSO only) or treated with 0.01 µM or 10 µM decitabine for four days. DNA was extracted and bisulfite converted, and PCR products were sequenced to analyze CpG methylation at each of the three individual CpG sites **(A–C)**. Plots show percentage methylation (n = 2 to 3 biological replicates). Statistical comparisons compare each CpG site in control cells with the same site in decitabine treated cells. **p < 0.01; ***p < 0.001.

Based on this information, we used PCR to amplify a 134 bp region immediately upstream of P1 using bisulfite modified DNA from control cells and cells treated for four days with decitabine at concentrations that do not change (10 nM) or that do change p63 isoform levels (10 µM). PCR products were sequenced with each primer to assess methylation changes at the three CpGs within the amplicon, designated as A, B, and C, from the most distal to the most proximal to the *TAP63* transcription start site. Under normal growth conditions, HaCaT, FaDu and SCC-25 cells showed high level cytosine methylation at all three CpG sites, with approximately 80% methylation at the distal CpG and > 90% methylation at the two sites closest to the transcription start site. High but not low dose decitabine for four days decreased methylation at all three CpG sites (**Figure 7**).

**Figure 8:**
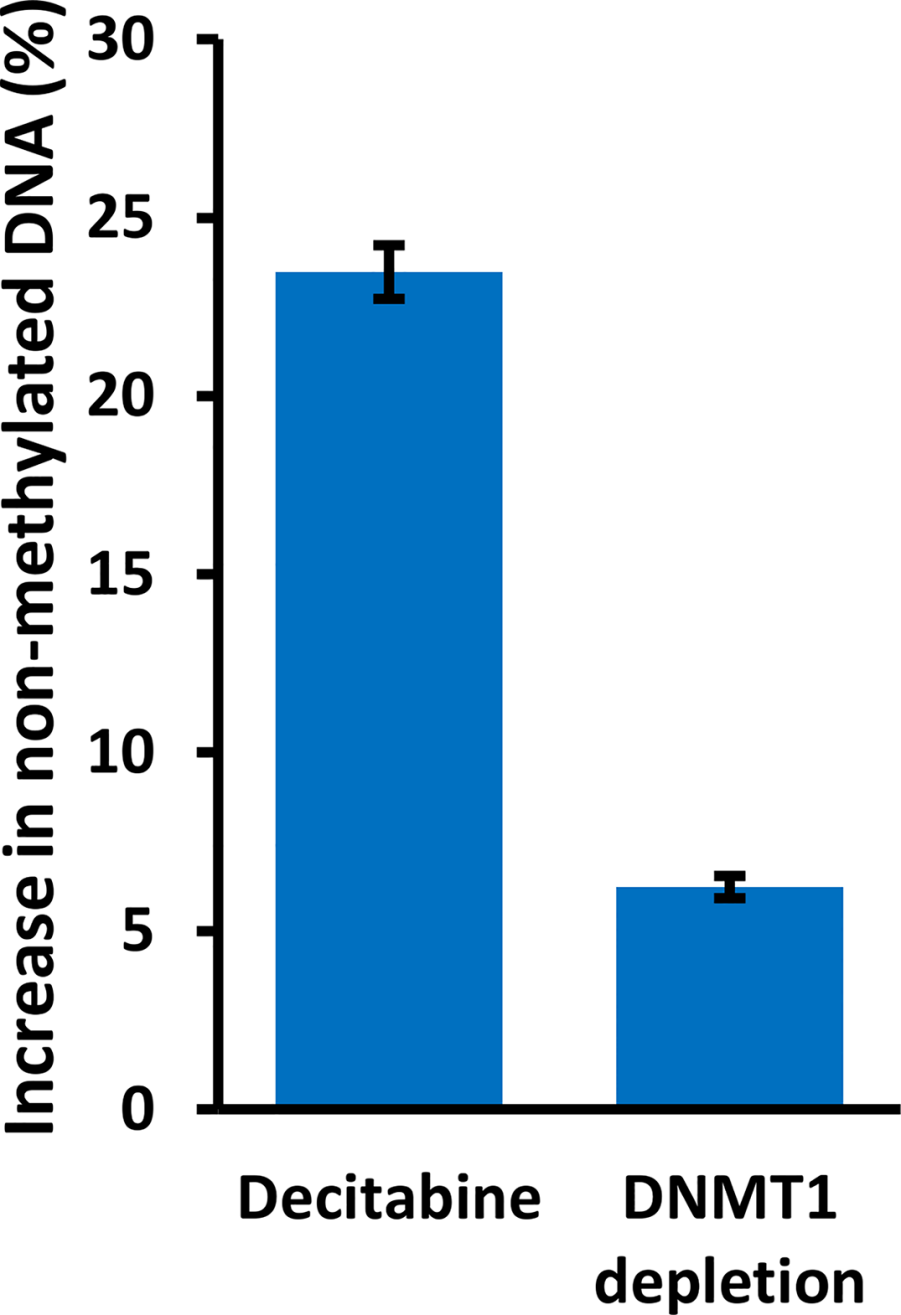
*DNMT1 reduces methylation at the TAp63 promoter but with a lesser effect than decitabine.* Parental HaCaT and FaDu cells were treated with 10 µM decitabine for four days, or *DNMT1*-shRNA cells were induced with 2 µg/ml doxycycline for six days. DNA was bisulfite converted and PCR products were sequenced to analyze the extent of CpG methylation at site A. The plot shows the percentage increase in non-methylated cytosine (average changes in HaCaT and FaDu combined) after treatment, compared to matched control cells without decitabine or doxycycline.

We also analyzed specific DNMT1 depletion by doxycycline treatment of HaCaT and FaDu *DNMT1*-shRNA cells and sequencing of the distal CpG site. Two independent *DNMT1*-shRNA clones were used for each cell line and the data are summarized in **Figure 8** by the average change of non-methylated CpG after doxycycline-mediated DNMT1 depletion for six days compared to the matched controls (*DNMT1*-shRNA HaCaT and FaDu cells without doxycycline). The magnitude of the effect of DNMT1 depletion is compared to the same average change in non-methylated CpG in parental HaCaT and FaDu cells after 10 µM decitabine for four days compared to DMSO only (derived from data in **Figures 7A, B**). *DNMT1*-shRNA caused an average 6.2% increase in demethylation in contrast to the average 23.5% increase in demethylation after decitabine (**Figure 8**).

### The TAP63 Promoter Is Highly Methylated in Primary SCC Tumor Samples

**Figure 9:**
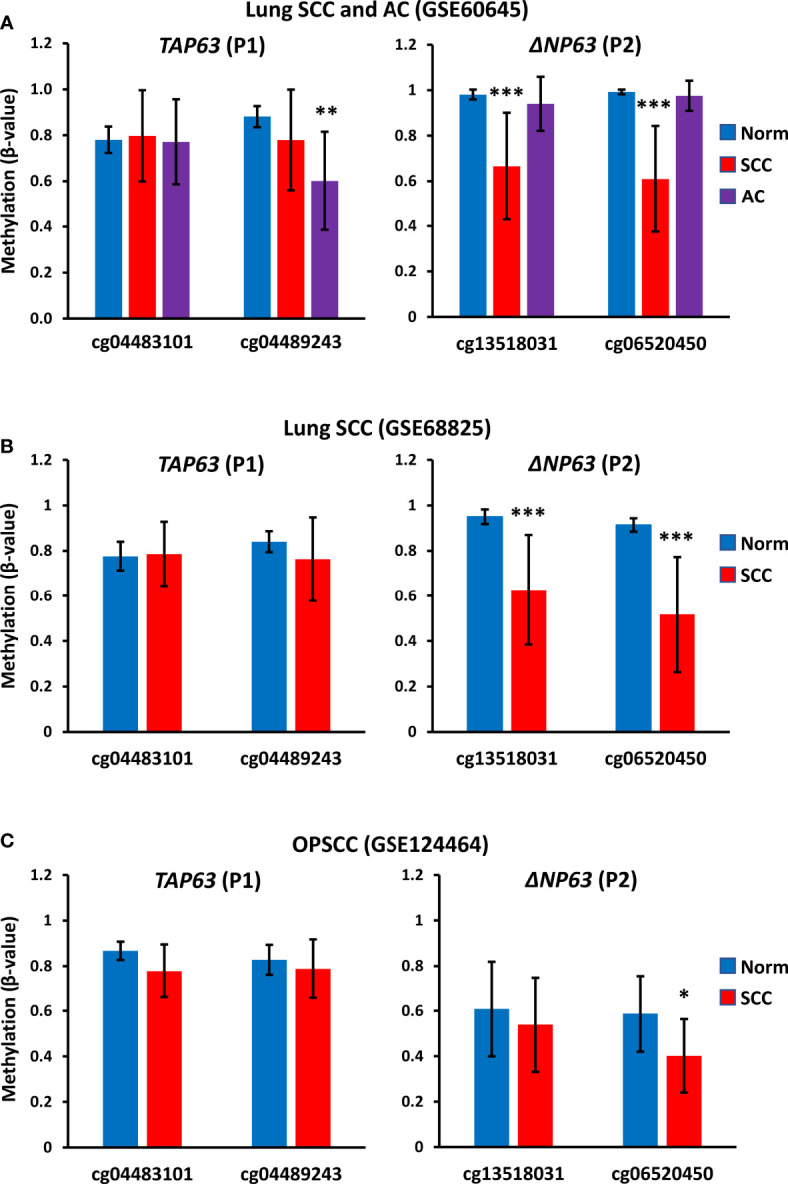
*TAP63 promoter methylation levels in primary SCC tumor samples*. Graphs show data for two probes (cg04483101 and cg04489243) that lie immediately upstream of the *TAP63* promoter (P1) (see Figure 6) and for two probes closest to the *ΔNP63* promoter (P2) identified in (25). **(A)** Data from GSE60645 containing normal lung (Norm; n=12), lung SCC (n=22) and lung adenocarcinoma (AC, n= 81). **(B)** Data from the cancer genome atlas analysis of lung SCC (GSE68825; normal lung (n=43) and lung SCC (n=96). **(C)** Data from GSE124464 of oropharyngeal SCC (OPSCC) (n=64) and normal oral mucosa (n=5). Graphs represent mean β-values ± SD. β-values range from 0 (no methylation) to 1 (complete methylation). *p < 0.05, ***p < 0.001, Mann-Whitney test.

To investigate whether methylation at the *TAP63* promoter is seen only in the cell lines studied here or is a common finding in clinical SCC samples, we also analyzed publicly available methylation data in three independent patient cohorts that included normal tissue samples and tumor samples (two datasets of lung SCC and one of oropharnygeal SCC (OPSCC). Analysis of the two CpG sites present on the array that lie immediately upstream of the *TAP63* transcription start site (cg04483101 and cg04489243) shows high level methylation in normal oropharyngeal mucosa and normal lung in all cohorts, and these values are not significantly altered in OPSCC or the lung SCC samples (**Figures 9A–C**). GSE60645 also contained 81 samples of adenocarcinoma (AC), showing maintained high level P1 methylation at cg04483101 but a decreased methylation for cg04489243 in AC compared to normal lung samples (**Figure 9A**). The levels of methylation at the two sites closest to the *ΔNP63* promoter (P2) previously reported to inversely correlate with ΔNp63 levels (25) are also provided (cg13518031 and cg06520450). Unlike P1 methylation, P2 methylation at both sites is decreased in lung SCC but is maintained at a high level in AC compared to normal lung (**Figures 9A, B**). These data are in keeping with observations that some lung AC show p63 positivity using pan-p63 antibodies, but are not positive with antibodies to ΔNp63 (1, 39), suggesting that lung AC may express TAp63 but not ΔNp63 due to altered DNA methylation at P1 but not P2. In the oropharynx, P2 promoter methylation levels are relatively low in normal tissue samples [reflecting the high level of ΔNp63 protein and mRNA levels in normal oral mucosa (1, 2, 16, 34)] and methylation at cg06520450 is further decreased in OPSCC (**Figure 9C**). These data strengthen and extend our findings of high *TAP63* P1 methylation *in vitro* in squamous cell lines used in our experimental studies, implying that clinical demethylation of these sites would similarly increase TAp63 activity in SCC patients.

## Discussion

Methylation of cytosine in CpG motifs is a major epigenetic modification that acts to repress gene transcription and is associated with chromatin compaction and inaccessibility. DNA methylation is carefully controlled during development and cell differentiation and is dysregulated in many cancers, where hypermethylation of tumor suppressor genes is a common finding to inhibit their expression (40–42). In SCC, genes that are commonly hypermethylated to repress their expression and tumor-suppressive activities include CDKN2A and RASSF1 (growth arrest), MGMT (DNA repair), and DAPK (apoptosis) (43, 44). In general, CpG methylation is initiated by DNMT3 enzymes, whereas DNMT1 maintains DNA methylation as cells divide. However, this is an over-simplification and it is known that DNMT3 may also maintain and/or remodel methylation patterns (45, 46). In addition, although promoter DNA methylation is associated with transcriptional silencing, DNA methylation in the gene body is associated with gene expression (47).

Here, we examined whether p63 variants, TAp63 and ΔNp63, are subject to regulation by DNA methylation. ΔNp63 is present at high levels in normal squamous cells and is often increased further in SCC, sometimes but not always due to chromosome 3 amplification, with an average more than 200-fold higher level of *ΔNp63* than *TAp63* mRNA in SCC (1, 25). This increase in *ΔNp63* mRNA is associated with hypomethylation at the P2 promoter and intronic sites closest to the *ΔNp63* transcription start site (23–25). In contrast, lymphomas and leukemias [that express TAp63 but not ΔNp63 (21, 48)] exhibit hypomethylation at P1 in association with increased *TP63* levels (21, 22). Therefore, we hypothesized that *TAP63* may be repressed and *ΔNP63* activated in squamous cells by differential promoter methylation. Reducing DNA methylation is then expected to de-repress *TAP63* transcription, allowing transcriptional activation of this tumor suppressor protein, but not to influence *ΔNp63* mRNA levels from the endogenously hypomethylated P2 regions.

**Figure 10:**
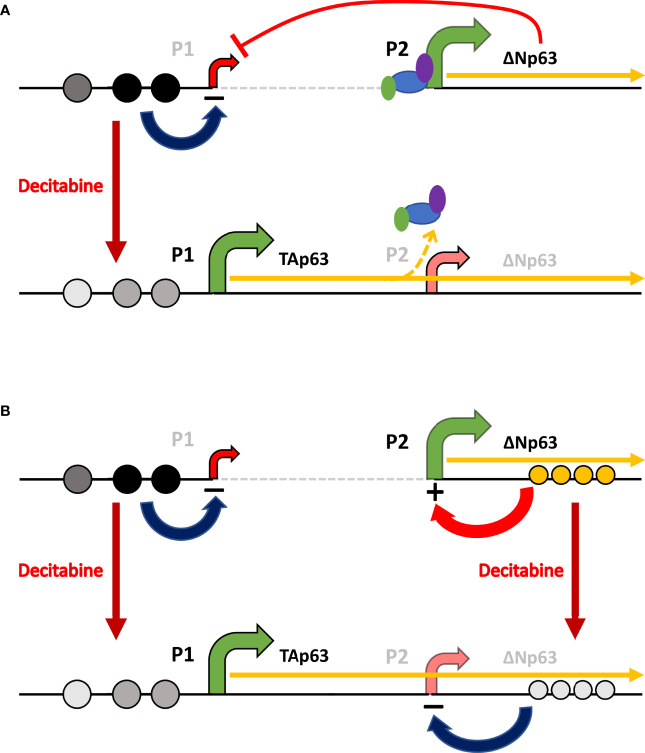
*Schematic of the potential mechanisms involved in reciprocal regulation of TAp63 and ΔNp63.* **(A)** In squamous cells, where ΔNp63 is highly expressed from the P2 promoter, methylation of CpG sites (dark gray or black circles) at P1 inhibits *TAP63* transcription and reduced methylation at these sites after decitabine (lighter gray circles) allows TAp63 production. As the *TAP63* mRNA is elongated, it unavoidably transcribes through the P2 region thereby reducing transcription initiation at this site, perhaps by displacing transcription factors and RNA Polymerase II (colored ovals). **(B)** In addition, methylation/hydroxy-methylation within the gene body (orange circles) may activate ΔNp63 transcription, which is reduced after decitabine.

In keeping with this concept, the universal demethylating agent decitabine (5-dAza) increased TAp63 protein and mRNA levels, with between 45- and 80-fold higher protein and up to 150-fold higher *TAP63* mRNA after decitabine. These changes were associated with decreased cytosine methylation at each of three CpGs lying immediately upstream of the *TAP63* transcription start site. At the same time, ΔNp63 protein and mRNA levels were reduced by decitabine, shifting the isoform ratio even further towards tumor suppression. The effects on *ΔNP63* mRNA are surprising and difficult to explain by demethylation at the *ΔNP63* gene promoter. One relatively simple explanation is that activating the upstream promoter hinders transcription from the downstream promoter, and transcription from the downstream promoter is more efficient in the absence of upstream promoter usage and transcriptional read-through at the downstream promoter. This mechanism takes into account the physical difficulty in transcribing through an active downstream promoter on the same gene if the downstream promoter is occupied by transcription factors, RNA polymerase and associated proteins and can explain how *TAP63* and *ΔNP63* mRNAs show inverse responses to methylation changes in SCC cells and the regulation of isoform-specific transcription in epithelial cells *versus* lymphocytes (**Figure 10A**).

An alternative explanation is that ΔNp63 acts as a transcriptional repressor of TAp63 (49), and that DNA damage caused by high concentrations of decitabine (36, 37) reduces ΔNp63 by ubiquitin-mediated degradation (50–52) to allow *TAP63* transcription. In addition to DNA damage as a cause of reduced ΔNp63 protein after decitabine, this agent is known to cause growth arrest and apoptosis along with caspase activation in malignant cells including SCC cells (53–55) (and see **Supplementary Figure S4** of cell growth data in our study), suggesting that these proteolytic pathways may contribute to the reduced ΔNp63 protein levels observed after decitabine. However, reduced protein levels of ΔNp63 by post-translational degradation through capases, the proteasomal pathway or any other mechanism does not directly account for transcriptional lowering of *ΔNP63* mRNA. In contrast, decitabine reciprocally changed *ΔNP63* and *TAP63* mRNA levels, indicating transcriptional regulation rather than protein degradation as at least part of the mechanism for reduced ΔNp63 protein levels. Moreover, although an increase in TAp63 was reported in one study of ΔNp63-specific knockout mice (49), this was not observed in another study using the same transgenic mice (56) or in a similar ΔNp63-specific knockout mouse (57). Thus, whilst downregulation of ΔNp63 protein by DNA damage or apoptosis may contribute to TAp63 induction, the evidence is weak. We also showed variable induction of DNA damage after decitabine, measured by γ-H2AX, and the level of damage did not correlate with the effects of decitabine on TAp63 or ΔNp63. In addition, the *DNMT1*-shRNA experiments indicate a direct effect of reduced methylation in causing reduced ΔNp63 protein levels.

Another potential explanation for decreased *ΔNP63* mRNA after decitabine is altered maintenance of DNA hydroxymethylation in enhancer regions (**Figure 10B**). DNMT3A promotes hydroxymethylation in the center of active enhancers, while DNMT3B promotes methylation across the gene body, and both are essential for *ΔNP63* transcription (58). Against this theory, hypomethylation at the P2 promoter is seen in SCC in association with ΔNp63 (23, 24), and hypomethylation of two intron sites nearest the ΔNp63 transcription start site is also associated with the levels of *ΔNP63* transcription in SCC (25). Thus, the available evidence indicates that demethylation at these sites increases ΔNp63, whereas we find that global demethylation, which would include demethylation at these sites, decreases ΔNp63. However, it is also important to note that DNA methylation is interrelated with histone methylation/acetylation, providing a further layer of complexity to epigenetic regulation (58–60). Additional investigations of DNA methylation/hydroxymethylation and histone methylation/acetylation profiles at the *ΔNP63* promoter, enhancers and gene body will be required to distinguish between these potential mechanisms of action.

Our experiments using *DNMT1* depletion with two *DNMT1*-shRNAs that effectively reduced DNMT1 levels showed that both constructs increased *TAP63* and decreased *ΔNP63* and *KRT5* mRNA levels, albeit with lesser changes than decitabine. These data provide independent validation for the role of DNA hypomethylation in activating TAp63 and repressing ΔNp63 in SCC. That the effect of DNMT1 depletion is lower than that of decitabine indicates that molecules other than DNMT1 are involved. This lower level of demethylation is an expected result, reflecting the continued presence of DNMT1 protein after mRNA inhibition together with the maintained activity of other DNMT enzymes that would not be operative in the presence of decitabine but are not inhibited by *DNMT1*-specific shRNA and are therefore able to maintain and/or renew methylation at previously modified CpGs to at least some extent (45).

The alternative hypotheses for reciprocal isoform regulation after demethylation with decitabine are not mutually exclusive and it may be that a combination of mechanisms is involved. Whilst the precise mechanism(s) involved in the inverse relationships of TAp63 and ΔNp63 in SCC is uncertain, it will be important to determine whether de-methylation induces TAp63 in all cancers, including tumor types lacking p63 expression but containing mutant p53, where TAp63 may replace p53 activity for therapeutic gain (10, 11, 13), or if TAp63 activation occurs only in cells with an already active *TP63* gene. These latter tumor types include not only SCC but also other common epithelial tumors such as breast, bladder and prostate that contain ΔNp63 either in the majority of cells or in the specific subset of cancer stem cells (1, 2, 8, 32, 61–63). In this respect, it has been reported that decitabine alters *TAP63* and *ΔNP63* mRNA levels in an inconsistent and non-reciprocal fashion in bladder cancer cell lines (64), different from our results in SCC. However, it must be noted that the findings of high level *TAP63* and absent *ΔNP63* in normal bladder cells are the opposite of a subsequent study showing abundant ΔNp63 mRNA and protein and absent or minimal *TAP63* mRNA and undetectable TAp63 protein in normal urothelial cells (65). Similarly, analyses of primary bladder cancer samples and a larger panel of bladder cancer cell lines also showed absent or extremely low levels of TAp63 protein and mRNA compared to ΔNp63 protein and mRNA, including some of the cell lines previously reported to contain abundant *TAP63* mRNA (65, 66), casting doubt on the validity of the findings after decitabine. Clearly, further experiments using the improved isoform-specific reagents now available will be required to determine the expression patterns of TP63 isoforms in this cancer type.

In conclusion, we have shown that inhibiting DNA methylation causes a switch in the relative levels of p63 isoforms, potentially converting ΔNp63-mediated tumor promotion and therapy resistance (7–9, 12, 13) towards TAp63-mediated tumor suppression (3, 4, 6, 10, 11). By identifying DNA hypermethylation at the *TAP63* promoter in SCC, these data add this gene to the list of tumor suppressors that are epigenetically silenced in malignancy (40–42). Moreover, our data indicate that demethylation at this locus simultaneously reduces transcription of the related ΔNp63 isoform, indicating the potential for additive effects in SCC. In addition to the direct effects of p63 isoform switching for cell growth/survival, reciprocal TAp63 and ΔNp63 regulation would be expected to further increase the response to cancer therapeutics compared to the effects of ΔNp63 reduction or TAp63 induction alone (9–11, 13, 67).

## Data Availability Statement

The original contributions presented in the study are included in the article/**Supplementary Material**. Further inquiries can be directed to the corresponding author.

## Author Contributions

ZP and PC designed the research approaches. ZP, VH and VT performed experiments. ZP, BV and PC analyzed and interpreted data. ZP and PC wrote the manuscript. All authors contributed to manuscript revision and approved the final version.

## Funding

This work was funded by the Czech Science Foundation (GACR 19-06530S), the European Regional Development Fund (ENOCH CZ.02.1.01/0.0/0.0/16_019/0000868), and the Ministry of Health, Czech Republic (MMCI 00209805).

## Conflict of Interest

BV is a consultant for Moravian Biotechnology.

The remaining authors declare that the research was conducted in the absence of any commercial or financial relationships that could be construed as a potential conflict of interest.

## Publisher’s Note

All claims expressed in this article are solely those of the authors and do not necessarily represent those of their affiliated organizations, or those of the publisher, the editors and the reviewers. Any product that may be evaluated in this article, or claim that may be made by its manufacturer, is not guaranteed or endorsed by the publisher.
