# Supplementary material for: DNA Demethylation Switches Oncogenic ΔNp63 to Tumor Suppressive TAp63 in Squamous Cell Carcinoma
Source: Front Oncol. 2022 Jul 14;12:924354. doi: 10.3389/fonc.2022.924354 (PMC9331744; doi:10.3389/fonc.2022.924354)
Supplement: Supplementary file 1 [file DataSheet_1.pdf]

Supplementary Material and Methods

Viability/Metabolic Activity Assay

Cell viability was determined using Resazurin sodium salt assay protocol (Sigma-Aldrich). HaCaT, FaDu or SCC25 cells were seeded into 96-well plates in their normal growth medium (5000 cells/well for HaCaT, 1250 cells/well for FaDu and 5000 cells/well for SCC-25). After overnight attachment, decitabine (5-dAza) was added at concentrations ranging from 0 to 2 μM and cells were incubated for a further 4 days. Resazurin was then added at a final concentration of 100 μg/ml and incubated for 3 h at 37 °C. Fluorescence was measured at 530 nm excitation and 590 nm emission using a fluorescence microplate reader (Tecan Infinite M1000 Pro). Each sample was measured in 8 technical replicates.

Methylation microarray analysis and cohort details

All data were originally produced using bisulfite converted DNA hybridized to Illumina HumanMethylation 450 BeadChip (platform GPL13534). Probes cg04483101 and cg04489243 were selected as the probes on the array that are closest upstream of the *TAP63* transcription start site, similar to the sequence we analyzed in cell lines. Processed microarray data were obtained from the GEO repository using GEO2R and specifying the individual probe set. The "Sample values" option was used to retrieve data that were saved in Excel for analysis. Sample annotation was added using the sample information files. GSE68825 contains TCGA data from 43 normal lung samples and 96 lung SCC. GSE60645 contains data on 132 lung samples, from which the 12 normal samples the 22 SCC samples and the the 81 adenocarcinoma samples were used (the remaining samples consisting of other tumor types were not used in the analysis). GSE 124644 contains 5 samples of normal oropharyngeal mucosa and 64 samples of oropharyngeal SCC (note that FaDu is an oropharyngeal SCC cell line). Each cohort was analyzed separately for the mean b-values of normal and SCC samples.

Western blotting

Cells were washed three times with cold PBS and lysed in 150 mM NaCl, 1% NP-40, 50 mM Tris pH 8.0, 5 mM EDTA pH 8.0 supplemented with 50 mM NaF, protease inhibitor cocktail and phosphatase inhibitors (Thermo Fisher Scientific, Waltham, MA, USA). After quantitation of total protein concentration, samples were diluted with 4x sample buffer (62.5 mM Tris-HCl pH 6.8, 2% SDS, 0.4% glycerol, 0.04% bromophenol blue, 5% β-mercaptoethanol) and heated at 95 °C for 5 min. Proteins were separated on 10% polyacrylamide gels and transferred to nitrocellulose membranes. Membranes were cut into upper and lower portions for ΔNp63, TAp63 or DNMT1, or for γ-H2AX or β-actin, respectively, and blocked for 1 h (ΔNp63, TAp63, DNMT1 and β-actin) or 2h (γ-H2AX) with 5% non-fat milk in PBS with 0.1% Tween-20 (PBS/T) at room temperature. To assess p63 variant proteins, we used mouse monoclonal antibodies that identify either ΔNp63 (clone ΔNp63-1.1, 2.4 μg/ml) or TAp63 (clone TAp63-4.1, 1.2 μg/ml). In addition to their specificity for ΔNp63 or TAp63, these antibodies do not cross-react with p53 or p73. Antibodies to DNMT1 (1:1000, clone D63A6, #5032, Cell Signaling Technology, Danvers, MA, USA), γ-H2AX (1:500, phospho-H2AX(Ser139), #9718; Cell Signaling Technology) and β-actin (0.2 μg/ml, clone C4, sc-47778, Santa Cruz Biotechnology, Dallas, TX, USA) were also used. Antibodies were applied overnight at 4°C, membranes were washed three times in PBS/T and once in PBS and then incubated with peroxidase-conjugated goat anti-mouse or anti-rabbit IgG (Jackson Immunoresearch, West Grove, PA, USA) diluted 1:5000 for 1 h at room temperature. After washing in PBS/T and PBS, bands were visualized using enhanced chemiluminescence (ECL, Amersham Pharmacia Biotech, Bucks, UK) and X-ray film. Scanned images were quantified by densitometry using ImageJ and normalized to actin.
