# Supplementary material for: DNA Demethylation Switches Oncogenic ΔNp63 to Tumor Suppressive TAp63 in Squamous Cell Carcinoma
Source: Front Oncol. 2022 Jul 14;12:924354. doi: 10.3389/fonc.2022.924354 (PMC9331744; doi:10.3389/fonc.2022.924354)
Supplement: Supplementary file 2 [file DataSheet_2.pdf]

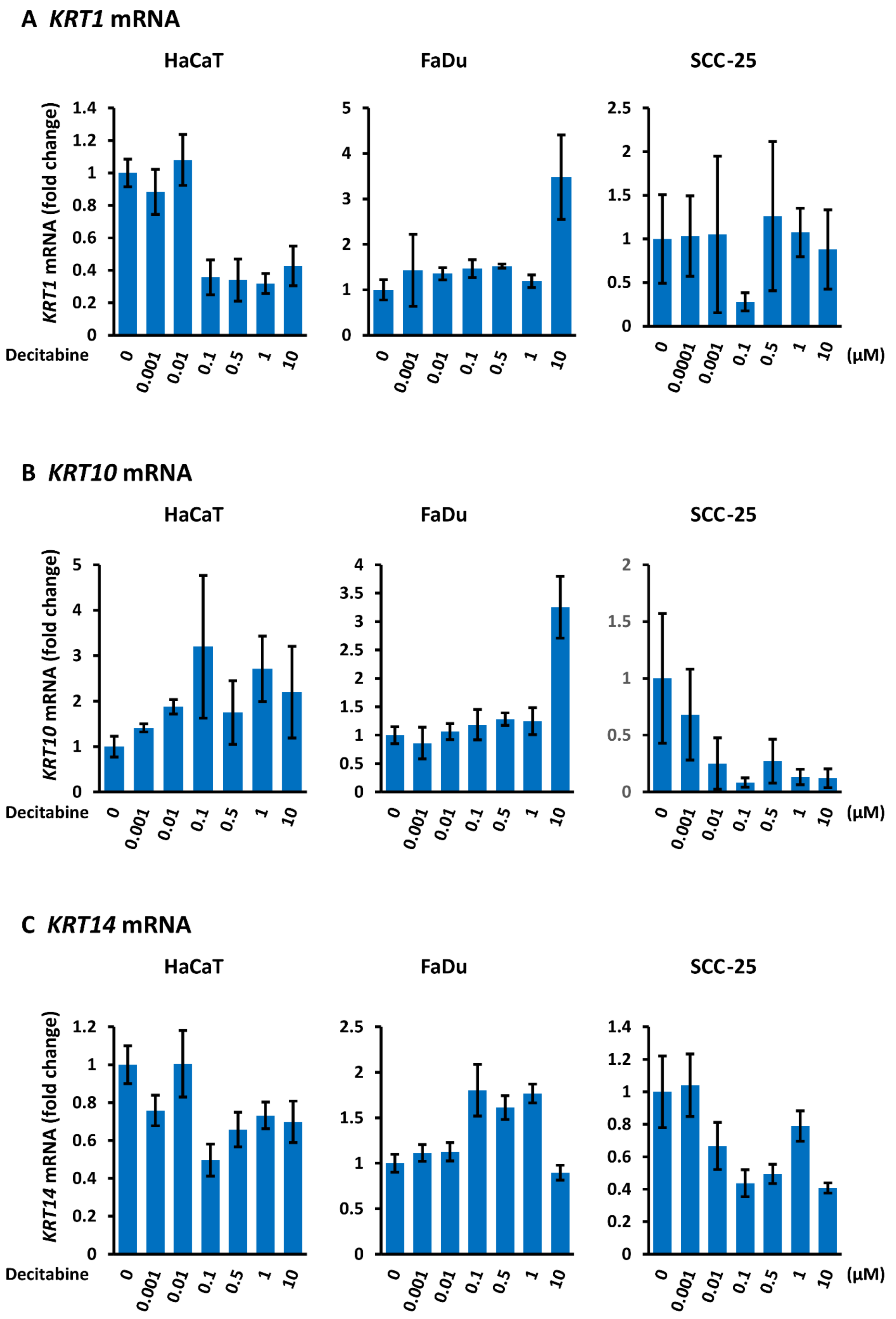

Supplementary Figure S1. *Changes in KRT1 and KRT10 and KRT14 mRNA after decitabine.* Cells were treated with the indicated concentrations of decitabine for four days and analyzed by RT-qPCR. Data are shown as fold change ± SEM compared to control cells (0; DMSO only) and are normalized to *ACTB* mRNA (n = 3 biological replicates with technical triplicates for each dose in each cell line).
