# Supplementary material for: DNA Demethylation Switches Oncogenic ΔNp63 to Tumor Suppressive TAp63 in Squamous Cell Carcinoma
Source: Front Oncol. 2022 Jul 14;12:924354. doi: 10.3389/fonc.2022.924354 (PMC9331744; doi:10.3389/fonc.2022.924354)
Supplement: Supplementary file 3 [file DataSheet_3.pdf]

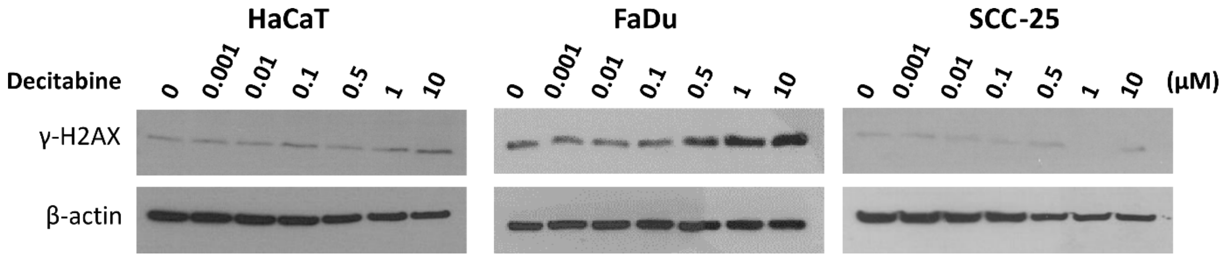

Supplementary Figure S2. *DNA damage after decitabine*. Cells were treated with the indicated concentrations (µM) of decitabine for 4 days and the levels of γ-H2AX were measured by Western blotting. 0; DMSO only. A representative blot is shown (n=3 biological replicates), with β-actin as loading control. (Note that the β-actin blot shown for FaDu is the same as used in Figure 1A, and that for SCC-25 is the same experiment shown in Figure 2A, where multiple proteins were measured using different strips of membrane from the same blot. HaCaT blots are from an additional experiment).
