# Supplementary material for: DNA Demethylation Switches Oncogenic ΔNp63 to Tumor Suppressive TAp63 in Squamous Cell Carcinoma
Source: Front Oncol. 2022 Jul 14;12:924354. doi: 10.3389/fonc.2022.924354 (PMC9331744; doi:10.3389/fonc.2022.924354)
Supplement: Supplementary file 4 [file DataSheet_4.pdf]

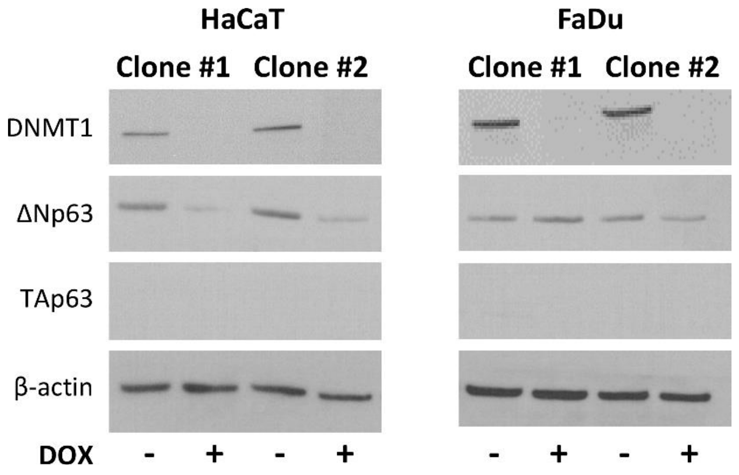

Supplementary Figure S3. DNMT1 depletion reduces ΔNp63. Western blotting of DNMT1, ΔNp63 and TAp63 in HaCaT and FaDu cells with or without doxycycline (DOX) induction of DNMT1 shRNA. Two independent clones are shown. β-actin is shown as loading control.
