# Supplementary material for: DNA Demethylation Switches Oncogenic ΔNp63 to Tumor Suppressive TAp63 in Squamous Cell Carcinoma
Source: Front Oncol. 2022 Jul 14;12:924354. doi: 10.3389/fonc.2022.924354 (PMC9331744; doi:10.3389/fonc.2022.924354)
Supplement: Supplementary file 5 [file DataSheet_5.docx]

**
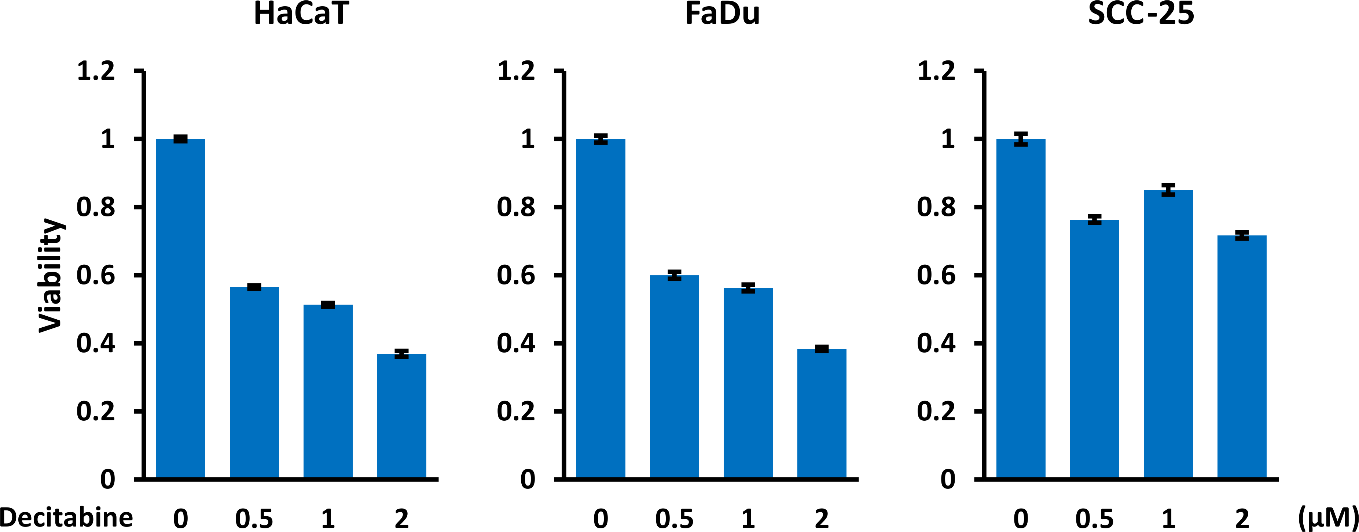
**

**Supplementary Figure S4.** *Decitabine reduces cell numbers*. Results of resazurin assays performed on HaCaT, FaDu and SCC-25 cells treated for four days with the indicated concentrations (μM) of decitabine. Data are normalized to untreated cells. Mean ± SD. All treated cells show significantly lower levels (p < 0.001 for all; n = 8 technical replicates for each concentration in each cell line).
