# Supplementary material for: DNA Demethylation Switches Oncogenic ΔNp63 to Tumor Suppressive TAp63 in Squamous Cell Carcinoma
Source: Front Oncol. 2022 Jul 14;12:924354. doi: 10.3389/fonc.2022.924354 (PMC9331744; doi:10.3389/fonc.2022.924354)
Supplement: Supplementary file 6 [file DataSheet_6.pdf]

Supplementary Table 1 *Primer sequences for bisulfite modified PCR and RT-qPCR.*

Primer	Sequence (5’ - 3’)
BSP forward	GAGGGGGAAGAATAATAGTAGAGAG
BSP reverse	CTCTCCTTCATACATATTTACAAAAAAAC
TAP63 forward	GTCCCAGAGCACACAGACAA
TAP63 reverse	TAGCATGGACTGTATCCGCA
ΔNP63 forward	AGCCAGAAGAAAGGACAGCA
ΔNP63 reverse	TCACTAAATTGAGTCTGGGCAT
KRT1 forward	ATGGACAACAACCGCAGTCT
KRT1 reverse	TGCAGCTCTTCATACTTGCTCT
KRT5 forward	CTCATGAACACCAAGCTGGC
KRT5 reverse	CTGCCACTGCCATATCCAGA
KRT10 forward	TCCCAACTGGCCTTGAAACA
KRT10 reverse	TGAGAGCTGCACACAGTAGC
KRT14 forward	ACCTCTCCTCCTCCCAGTTC
KRT14 reverse	TCGZGCACATCCATGACCTT
ACTB forward	GCCGACAGGATGCAGAAGGAG
ACTB reverse	CTAGAAGCATTTGCGGTGGAC

BSP, bisulfite sequencing PCR primer; red bases represent bisulfite converted residues (C->T; G->A). All other sequences were used for RT-qPCR.
